# Microwave Breast Imaging Using Compressed Sensing Approach of Iteratively Corrected Delay Multiply and Sum Beamforming

**DOI:** 10.3390/diagnostics11030470

**Published:** 2021-03-08

**Authors:** Mohammad Tariqul Islam, Md Tarikul Islam, Md Samsuzzaman, Salehin Kibria, Muhammad E. H. Chowdhury

**Affiliations:** 1 Department of Electrical, Electronic and Systems Engineering, Universiti Kebangsaan Malaysia, Bangi, Selangor 43600, Malaysia; p94299@siswa.ukm.edu.my (M.T.I.); kibriasalehin@gmail.com (S.K.); 2 Department of Computer and Communication Engineering, Patuakhali Science and Technology University, Dumki, Patuakhali 8602, Bangladesh; sobuz@pstu.ac.bd; 3 Department of Electrical Engineering, Qatar University, Doha 2713, Qatar

**Keywords:** microwave imaging, compressed sensing, delay multiply and sum, breast imaging, iterative correction

## Abstract

Microwave imaging (MI) is a consistent health monitoring technique that can play a vital role in diagnosing anomalies in the breast. The reliability of biomedical imaging diagnosis is substantially dependent on the imaging algorithm. Widely used delay and sum (DAS)-based diagnosis algorithms suffer from some significant drawbacks. The delay multiply and sum (DMAS) is an improved method and has benefits over DAS in terms of greater contrast and better resolution. However, the main drawback of DMAS is its excessive computational complexity. This paper presents a compressed sensing (CS) approach of iteratively corrected DMAS (CS-ICDMAS) beamforming that reduces the channel calculation and computation time while maintaining image quality. The array setup for acquiring data comprised 16 Vivaldi antennas with a bandwidth of 2.70–11.20 GHz. The power of all the channels was calculated and low power channels were eliminated based on the compression factor. The algorithm involves data-independent techniques that eliminate multiple reflections. This can generate results similar to the uncompressed variants in a significantly lower time which is essential for real-time applications. This paper also investigates the experimental data that prove the enhanced performance of the algorithm.

## 1. Introduction

Microwave imaging (MI) has been verified to be a reliable health monitoring technique that can play a prime role in diagnosing anomalies in breast tissue. A microwave transceiver can recognize small signal variations with the changes in the electrical properties of human tissues. Recent advances in microwave imaging show that it can be a viable solution as preclinical detection tools in human body imaging [1,2,3,4]. The on-phantom experiments shown in [4] describe the functionality of a microwave sensor for the successful localization of the target. The iterative reconstruction is referred to as post-processing of the acquired data. The clinical realization of MI is discussed with recent advancements in [3], where the MI technology is selected as a complementary modality of the existing screening systems for breast cancer. The potential role of MI and the clinical advances of this imaging is discussed in [1,2] where MI is described as a rapid and inexpensive method of diagnosis as compared to others.

The reliability of biomedical imaging-based diagnosis is substantially dependent on the imaging algorithm. There are three categories of MI, active, passive and hybrid [5]. Beamforming is the primary part of image reconstruction of active radar-based MI [6,7]. Among the two types of beamformers, data-independent beamformers take less time in image reconstruction while the data-adaptive beamformers have excessive computational complexity but produce better images [8].

The differential microwave diagnosis for breast [9], DMAS image reconstruction algorithm [10] and DAS algorithm have been suggested to improve imaging performance like the robust, interference-canceling, balanced deblurring. Several data-independent algorithms were utilized for microwave-based image processing, like Tissue Sensing Adaptive Radar (TSAR) [11] and confocal microwave diagnosis algorithm [12,13]. However, DAS and its variations are much more popular owing to their robust performance and simplicity. Most of these algorithms are applied to the received signal for the reconstruction of breast interior, feature extraction and post-processing to locate abnormalities. Another data-independent beamformer is DMAS which provides improved contrast and resolution in the reconstructed images by trading the complexity of computation. Several variants of DAS were introduced besides DMAS, including coherence factor DAS [14], improved DAS [15], and the iterative variant of DAS [16]. The improvements of DMAS over DAS has been proven in [13,17]. The iterative variant of DAS and DMAS is proposed in [18] where the authors removed the background clutter in the reconstructed images. The reconstructed images of the DMAS algorithm were used to detect the tumor after normalizing the maximum scatterer [19]. The compressed sensing approach has been reported in several image reconstruction techniques in MI [20,21]. A Bayesian CS approach to MI of an inhomogeneous object based on mesh discretization and electric field integral equation in the imaging region was proposed [22]. The CS was applied to separable surrogate functionals (SSF) optimization to reduce the sampling data volume in [23]. None of them is applied to the most popular DAS or variants of DAS algorithms. An iterative three-dimensional non-linear inverse scattering technique was proposed for the reduction of computational time using a nondispersive material model [24].

In this paper, a compressed sensing iterative variant of the DMAS algorithm is introduced for increasing the performance and reducing the runtime for microwave imaging applications using iteratively corrected DMAS beamforming. The proposed CS-ICDMAS works on the basis of computing all the channel power and eliminating comparatively lower power channels. This reduces the number of observations and saves the calculation time without decreasing the image quality. The practical imaging was tested, and the numerical analysis is also presented.

## 2. Imaging System Setup

**Figure 1:**
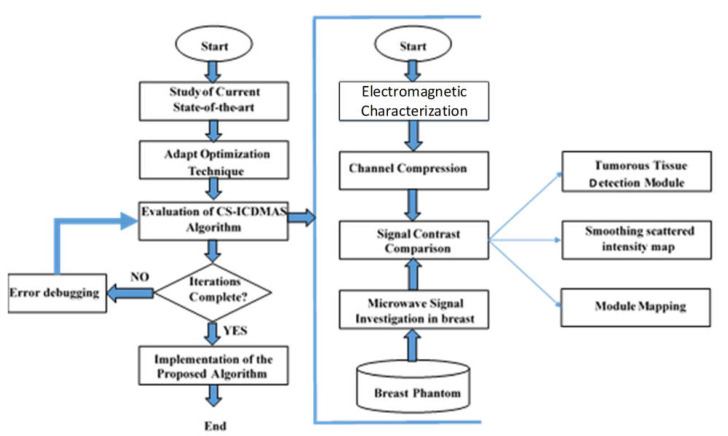
Flowchart of the research methodology.

**Figure 2:**
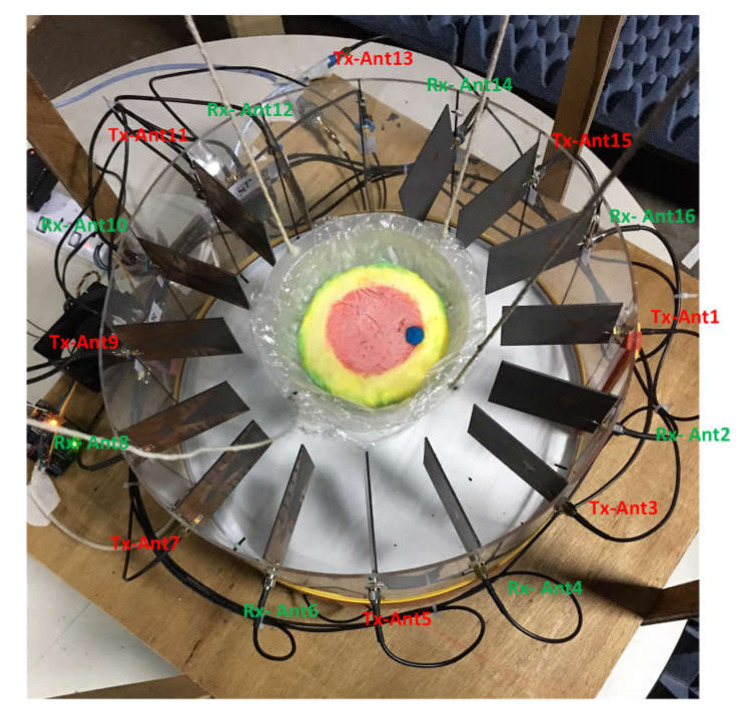
Imaging system setup (data-taking mode).

The detailed flowchart of the research methodology is presented in Figure 1. The imaging system consisted of a total of 16 antenna arrays with a mechanical rotating platform. A set of heterogeneous phantoms was tested. The system setup and phantom fabrication procedures can be found in the literature [15]. The system comprised eight transmitters and eight receiver antennas. A rotating turntable held the antenna array and data were taken using a MATLAB-based image processing platform using a vector network analyzer (VNA). The turntable rotated 360 degrees at a step of 7.2 degrees that resulted in 50 equal datapoints. As a result, the total number of channels was 8 × 8 × 50. Figure 2 shows the imaging system setup while collecting data from the system in the presence of a breast phantom.

### Antenna Performance Analysis

**Figure 3:**
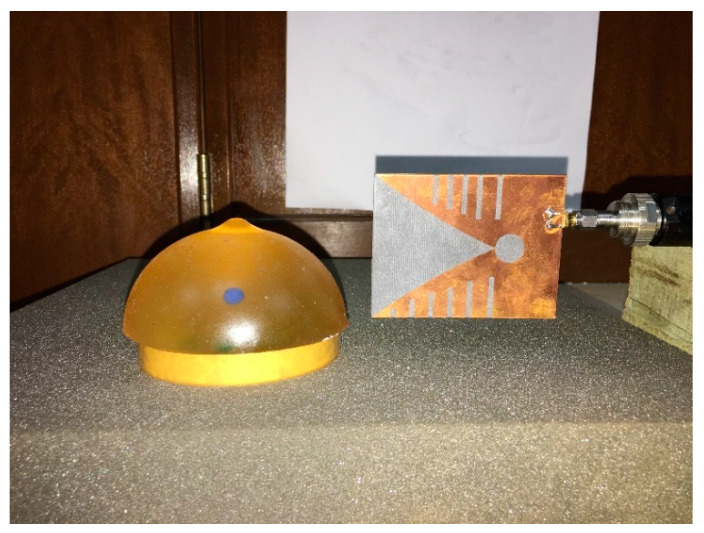
Reflection coefficient measurement with a phantom load.

**Figure 4:**
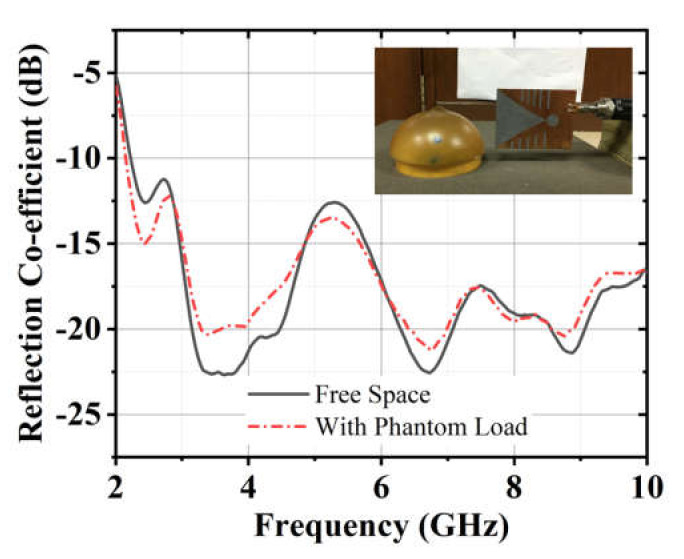
Measured reflection coefficient in free space and a phantom load.

**Figure 5:**
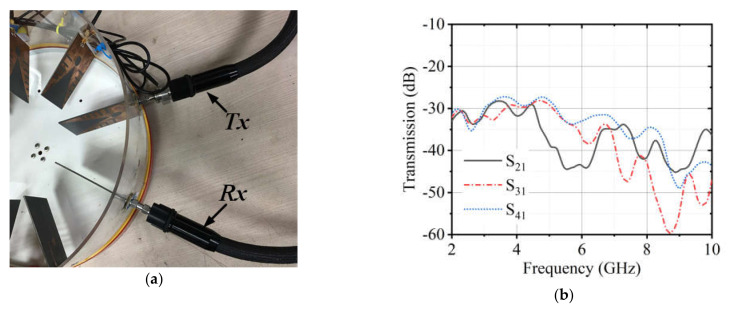
(**a**) Mutual coupling measurements set up with adjacent antennas (**b**) Transmission results.

The antenna used in the proposed system is a modified Vivaldi antenna. The design evaluation of the antenna can be found in [17]. The antenna has dimensions of 77.72 mm × 60 mm and a bandwidth from 2.70–11.20 GHz. The reflection coefficient is considered in free space and the heavy load of the phantom in this work. The setup for taking measurements in the presence of a phantom is shown in Figure 3. Figure 4 illustrates the measured reflection coefficient in free space and a phantom load. It was observed that the antenna could firmly maintain its bandwidth in both scenarios with minimal distortions. The mutual coupling between the adjacent antennas was also investigated. Figure 5a shows the setup of Tx and Rx antennas considered for measuring the isolation between the adjacent antennas. The results are presented in Figure 5b It was observed that the antennas had sound isolation and were maintained between −26 dB and −60 dB.

## 3. Beamforming Method

The proposed algorithm was analyzed based on the microwave signal contrast. The scattered microwave signals from healthy and unhealthy breast phantoms were compared. First, microwave signal contrast comparison took place between the reference microwave signal using numerical simulation of the full-wave time domain model and the scattered signal from the computational phantom. The successive approximation method was obtained to get the exact position and size of the tumor. There was no coupling medium between the phantom and the antenna; as a result, we needed to eliminate skin reflections. Thus, for artifact removal, the rotation subtraction was applied, which relied on the comparison between the original scattering and at least a single-rotated scattering [15]. The array of antennas is placed around the Region of Interest (ROI) in such systems. The array was rotated once, and the offset data were collected and recorded as a reference of initial radiance.

### 3.1. Channel Compression on Skin Reflection Removal

Different methods of artifact removal including rotation subtraction [25] and singular value decomposition (SVD) were proposed [26]. In the rotation subtraction method, it was assumed that the tumor was out of the axis of the rotation and the response received from the tumor would be well-preserved even after the subtraction of the scattered rotated position signals. In the SVD method, the breast shape is restricted as uniform and submerged in a coupling medium and the antenna array is maintained at a certain distance from the skin. In this work, the artifact removal was performed using the rotation subtraction method. The antenna array completed full rotation surrounding the phantom at a 7.2˚ step with a total of 8 × 8 × 50 channels. The equally sliced datapoint was denoted *Nφ* = 50. The system collected complex S-parameters in the frequency domain, S (*f, t_x_, r_x_, φ*), where f is the frequency, *t_x_* = 1, 3, 5, 7, 9, 11, 13, 15 and *r_x_* = 2, 4, 6, 8, 10, 12, 14, 16 are the transmitting and receiving antennas, respectively, and the alignment of the rotating platform angle is *φ*. A total of 201 datapoints was recorded for each rotation within the band from 2.7 to 8.0 GHz. Once the data were recorded for the original illumination, the array s rotated around the phantom to get offset data. The difference of the data from the different illuminations was computed to remove the effect of the skin.

In this work, the sample *S (f, t_x_, r_x_, φ)* was parted into two matrices based on *φ* becoming odd and even, or *S_odd_(f,t_x_,r_x_,φ_odd_)*, and *S_even_(f,t_x_,r_x_,φ_even_),* individually, where *φ_odd_* = odd number of datapoints (1, 3, 5,…N*_φ_*-1) and *φ_even_* = even number of data points (2, 4,6,…N*_φ_*). Thus, *S_odd_* can be considered initial illumination and *S_even_*—“*offset*” illumination. Finally, rotation subtraction was implemented by simply calculating the difference between the two matrices according to Equation (1):

(1) $$S_{skin\_removed}(f,t_{x},r_{x},\varphi_{odd})=S_{odd}(f,t_{x},r_{x},\varphi_{odd})-S_{even}(f,t_{x},r_{x},\varphi_{even})$$

**Figure 6:**
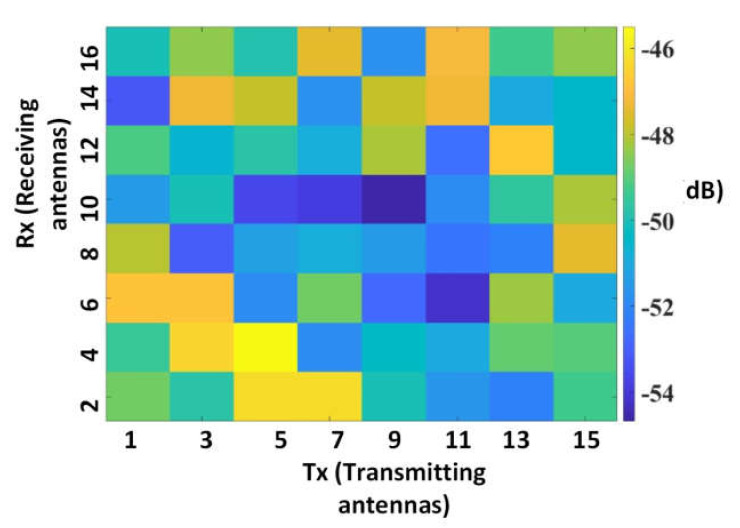
The 8 × 8 scattering matrix measured in the presence of a breast phantom.

The power of all the channels was calculated. The low-power channels were subtracted based on the compression factor ϗ. The measured 8 × 8 scattering matrix in the presence of a breast phantom is illustrated in Figure 6. The difference between the high- and low-power channels was visible. Afterwards, the total skin reflection data (R_skin_removed_) were calculated based on Equation (2):

(2) $$R_{skin\_removed}(t_{x},r_{x},\varphi_{odd})=\sum_{\forall f}S_{skin\_removed}(f,t_{x},r_{x},\varphi_{odd})$$

ϗ × n *(R_skin_removed_)* channels with the highest power were considered for the imaging from *S_skin_removed_* to produce compressed data as *S_compressed_(f,t_x_,r_x_, φ_odd_)*, where *n(R_skin_removed_)* is the number of channels and ϗ is the compression factor of 0 ≤ ϗ ≤ 1. The lower power channels were eliminated to reduce the execution time and make the process faster. After adjusting the skin reflections, the signals were converted to the time domain using the inverse Fourier transform to create Γ(t, t_x_, r_x_, φ_odd_). Then, the data in the Γ(t, t_x_, r_x_, φ_odd_) were processed via the delay multiply and sum (DMAS) algorithm for the reconstruction of the image [27,28]. Normally, DAS-based methods struggle in noisy settings where multiple reflections from various scattering sources need to be considered. In that case, DMAS outperforms DAS by generating sharper contrast images by utilizing the correlation process [29]. However, an earlier study reported that DMAS struggles in high-noise settings as well as in multiple target situations [30]. Therefore, in this research work, after applying compressed sensing (CS), we revised the typical DMAS algorithm by implementing iterative correction of the delay calculation. The correction was done by several iteration processes until convergence to the desired level of accuracy.

### 3.2. Compressed Sensing in DMAS

Three-dimensional Cartesian coordinates of all points inside the region of interest were represented in the *i* by 3 matrices, *C*, where *i* = 1,2,…, and *I* is the total number of points. Then, the *I* was generated from *C* by the *I* matrix, *P_C-C_*, containing the Euclidian distances between each possible pair of points in the imaging domain. Matrices *A_Tx_* and *A_Rx_* contained the optimized three-dimensional Cartesian coordinates of the transmitting and receiving antennas, respectively. Since the imaging domain was stationary, the antennas changed their distance from the points to be reconstructed as they rotated. Thus, *A_Txφodd_* and *A_Rxφodd_* were generated by determining all the antenna positions considered in the original orientation. Then, *P_Txφodd-C_* and *P_C-Rxφodd_*, containing the distances from each point to the transmitting and receiving antennas, were calculated from *C*, *A_Txφodd_*, and *A_Rxφodd_*. Finally, the delays needed for focusing each channel to focus on point *i* in *C* were evaluated by summing the distances of the transmitting and receiving antenna positions concerned to the point being focused. The wave propagation velocity in the background medium, air in this study (theoretically, speed of light c), was divided by the total distance to yield the appropriate delay, *τ* (*i*, *rx*, *φ_odd_*), as indicated in Equation (3):

(3) $$\tau (i,t_{x},r_{x},\varphi_{odd})=\frac{\sqrt{\varepsilon_{b}}(P_{Tx\varphi_{odd}-C}(i,t_{x},l)+P_{C-Rx\varphi_{odd}}(i,r_{x},l))}{c}$$

where *ε_b_* is the dielectric constant of the coupling medium. The delay *τ* was calculated from the estimated shortest distance and the reflected signal from *C*(*i*), which are the straight-line Euclidean distances calculated previously. The delays were further added to the signals for delivering a proper delayed signal. After multiplying by the paired delayed signal, they were summed for determining the scatter intensity at the allotted point in the ROI as shown in Equation (4). The paired signal multiplication compensated coherent signals together with higher values and hence improved the performance of DMAS over DAS [15,27].

(4) $${ϒ}_{DMAS}^{}(i)=\int_{-\infty }^{\infty }\sum_{\varphi_{odd}=1}^{N/2}\sum_{tx=1}^{Tx}\sum_{rx=1}^{Rx}\sum_{{\varphi^{\prime }}_{odd}=\varphi_{odd}}^{N/2}\sum_{tx^{\prime }=tx}^{Tx}\sum_{rx^{\prime }=rx+1}^{Rx}\left[\begin{array}{l} \Gamma (t-\frac{\tau (i,t_{x},r_{x},\varphi_{odd})}{\Delta t},t_{x},r_{x},\varphi_{odd})\\ \times \Gamma (t-\frac{\tau (i,t_{x}^{\prime },r_{x}^{\prime },{\varphi^{\prime }}_{odd})}{\Delta t},t_{x}^{\prime },r_{x}^{\prime },{\varphi^{\prime }}_{odd}) \end{array}\right]dt$$

### 3.3. Iterative Correction of CS-DMAS

The dielectric materials diminish the propagation speed; this results in increased time delay. Thus, the higher value of ϒ in the *C* region can be implied as to the higher dielectric region. The additional time adjustment can be made by properly increasing the distance while calculating *τ*. Here, we introduce an iterative approach to estimate the best-fitted delay as well as the scattered intensity map (SIM) evaluation. At the same time, a greater estimation of SIM is dependent on the proper adjustment of *τ*. However, the iterative process can lead to vulnerability and noise-sensitivity if we use ϒ directly. An inverse distance weighted integral averaging was introduced to produce a smoothed SIM, ϒ*’(i).* This method can be considered analogous to applying the 3D Green’s function in the distorted Born iterative method.

(5) $${ϒ}^{\prime }(i)=\int_{C}^{}\frac{{ϒ}_{DMAS}^{n-1}(i)}{1+P_{C-C}(i,j)}dj$$

An extra time, *P_C-C(i,j)_*, was adjusted by appropriately increasing the distances.

Then, the following equation was used to calculate modified delay:

(6) $$\tau^{\prime }(i,t_{x},r_{x},\varphi_{odd})=\tau (i,t_{x},r_{x},\varphi_{odd})+\frac{{ϒ}^{\prime }(i)}{c}$$

Here, *c* is the speed of light.

(7) $${ϒ}_{DMAS}^{}(i)=\int_{-\infty }^{\infty }\sum_{\varphi_{odd}=1}^{N/2}\sum_{tx=1}^{Tx}\sum_{rx=1}^{Rx}\sum_{{\varphi^{\prime }}_{odd}=\varphi_{odd}}^{N/2}\sum_{tx^{\prime }=tx}^{Tx}\sum_{rx^{\prime }=rx+1}^{Rx}\left[\begin{array}{l} \Gamma (t-\frac{\tau^{\prime }(i,tx,rx,\varphi_{odd})}{\Delta t},tx,rx,\varphi_{odd})\\ \times \Gamma (t-\frac{\tau^{\prime }(i,tx^{\prime },rx^{\prime },{\varphi^{\prime }}_{odd})}{\Delta t},tx^{\prime },rx^{\prime },{\varphi^{\prime }}_{odd}) \end{array}\right]dt$$

The new set of delays was used to reconstruct the SIM. Finally, the completion benchmark was inspected for convergence. The iteration of Equations (7) and (8) was evaluated for n = 1, 2,…, 7.

(8) $$E_{ϒ}=\sum {}_{\forall i}\left|{ϒ}_{DMAS}^{n}\right.-\left.{ϒ}_{DMAS}^{n-1}\right|$$

The iteration was terminated when E_ϒ_ decreased the expected level of accuracy after achieving convergence. In this paper, E_ϒ_ was < 10^−5^.

## 4. Imaging Results

The simulation of DMAS was performed on an HP Workstation Mid 2018 with a 3.7 GHz Intel Xeon E3-1245 processor and 32 GB 2400 MHz DDR4 RAM.

### 4.1. Contour Images

Two sets of the lab-made heterogeneous phantom were used to evaluate the performance of the proposed beamformer. The first one had a single tumor inside (phantom 1) and another one was designed with two tumors (phantom 2). The heterogeneous phantoms 1 and 2 had four layers of skin, fat, gland and tumor to be as realistic as possible [31]. The relative permittivity and conductivity of the skin layer were 24 and 2.49 S/m. The permittivity and conductivity of the fat layer were 6 and 1.72 S/m. The gland layer had the permittivity and conductivity of 16 and 3.27 S/m. Last of all, the tumor layer had relative permittivity and conductivity of 64 and 4.98 S/m. All the data were presented at 4 GHz. The backscattered signals were collected using an array of 16 Vivaldi antennas surrounding the phantom using the Agilent N5227 VNA. A total of 3200 (8 × 8 × 50) observations were taken from 2.5 to 8 GHz. The computation was done for DMAS and CS-DMAS with several compression factors.

**Figure 7:**
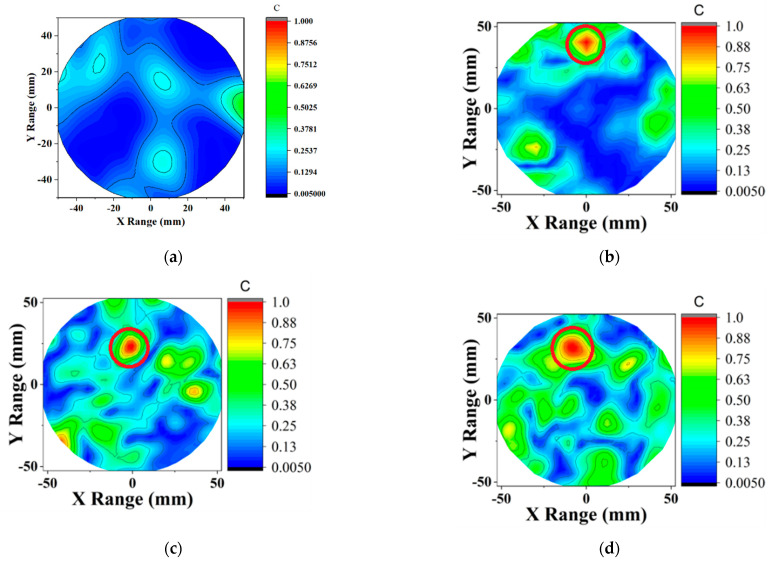
Contour plot of the reconstructed image of a single tumor phantom (**a**) without tumor, using (**b**) DMAS (left), (**c**) proposed CS-ICDMAS (50% compression) and (**d**) CS-ICDMAS with 75% compression.

**Figure 8:**
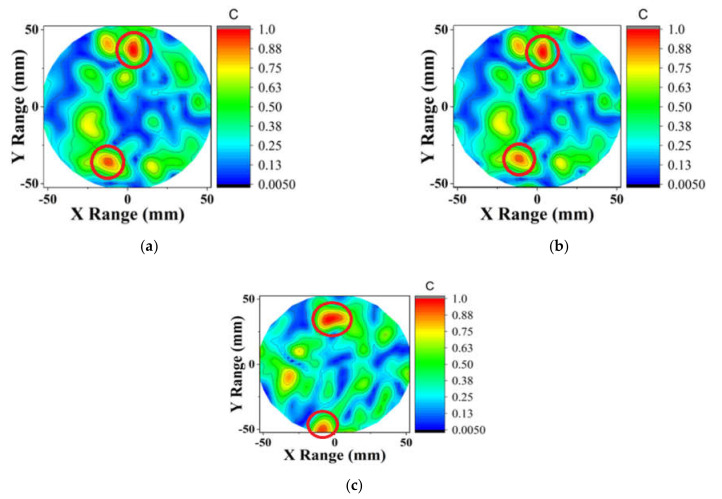
Contour plot of the reconstructed image of two tumor phantoms using (**a**) DMAS, (**b**) proposed CS-ICDMAS (50% compression), (**c**) CS-ICDMAS with 75% compression.

The reconstructed contour plot images are presented in Figure 7 and Figure 8 for phantom 1 and phantom 2, respectively. For the phantom 1 screening, DMAS successfully detected the tumor. After applying the compression to half of the observations ϗ (1600) and applying the Born iterative method, DMAS was still able to detect the target. The iteration converged after seven trials resulting in little delay variations. Thus, the location of the tumor was inconsistent, but the results are still promising as a screening tool. Figure 7c and Figure 8c show the image of CS-ICDMAS after compressing the observations of one-fourth of the total channel ϗ (800). It still identified the tumor in a red circle with some noise saving execution time and computational complexity.

### 4.2. Numerical Imaging Data

The numerical assessments in terms of signal-to-mean ratio (SMR) and execution time is described in this section. The SMR states the proportion of the maximum tumor energy to the mean energy from backscattered signals at a similar sample [32].

(9) $$SMR=\frac{Maximum\_Tumor\_Energy}{Mean\_Energy}$$

**Figure 9:**
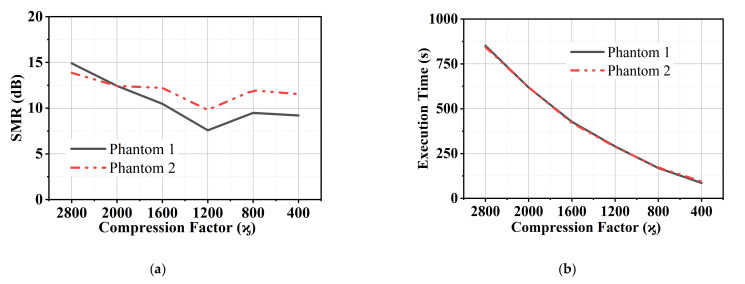
(**a**) SMR and (**b**) execution time with respect to the compression factor.

Numerical imaging data in terms of SMR and execution time are shown in Table 1. The graphical representation of the signal to mean ratio and execution time against the compression factor is presented in Figure 9a,b. The SMR of CS-ICDMAS remains decreased with the compression factor. The SMR shows a proportional relation with the execution time. But the drop of SMR concerning execution time is not huge. The proposed algorithm shows exponential improvement in reducing execution time by maintaining image quality and detection. These results validate the capability of CS-ICDMAS to decrease computation time and reduce computational complexity by eliminating the weak channels by preserving detection performance. A comparison of the execution time for the DAS, CF (Coherence Factor)-DAS, DMAS and ICDMAS with the proposed method is provided in Table 2. It is noted that the proposed algorithm can reduce the time required for computations and simultaneously gives similar imaging output.

## 5. Conclusions

This work introduces a new compressed sensing approach of iteratively corrected delay multiply and sum beamforming over the well-known DMAS in terms of microwave imaging of the breast. The main disadvantage of conventional DMAS is the high computation load and runtime. An array of 16 antennas was used to receive the backscattered microwave signal from a breast phantom. The compressed sensing approach was proposed by finding low-power channels across all the observations and eliminating them based on the compression factor. Then, an iterative approach was applied till convergence. The artifact removal was performed using the rotation subtraction method. Imaging data from the experimental system were tested with the proposed approach and the reconstructed image and numerical analysis were presented. The proposed CS-ICDMAS algorithm shows exponential improvement in reducing execution time as compared to DAS, CF-DAS, DMAS and ICDMAS by maintaining image quality and detection which validate the capability of CS-ICDMAS to decrease computation time and reduce computational complexity by eliminating the weak channels and preserving detection performance.

## Figures and Tables

**Table 1:** Signal-to-mean ratio and execution time against the compression factor.

ϗ	SMR (dB)		Execution Time (s)	
	Phantom 1	Phantom 2	Phantom 1	Phantom 2
3200	15.20	14.87	1455	1440
2800	14.90	13.86	852	843
2000	12.43	12.42	619	620
1600	10.47	12.21	427	421
1200	7.58	9.80	290	287
800	9.48	11.95	169	172
400	9.20	11.53	86	95

**Table 2:** Comparison of the execution time with the methods presented in the literature.

Method	Execution Time(s)
DAS	2087
CF-DAS	2175
DMAS	2425
ICDMAS	1455
CS-ICDMAS (50% compression)	427
CS-ICDMAS (75% compression)	169

## Abbreviations List

CS	Compressed sensing
CF	Coherence Factor
CS-ICDMAS	Compressed sensing using iteratively corrected DMAS
DAS	Delay and sum
DMAS	Delay multiply and sum
MI	Microwave imaging
ROI	Region of interest
SIM	Scattered intensity map
SMR	Signal-to-mean ratio
SSF	Separable surrogate functionals
SVD	Singular value decomposition
TSAR	Tissue Sensing Adaptive Radar
VNA	Vector network analyzer
